# Outcomes of retroperitoneal fibrosis-related hydronephrosis and its risk factors for poor prognosis: a multi-center retrospective cohort study in Chinese patients

**DOI:** 10.3389/fmed.2024.1435870

**Published:** 2024-12-09

**Authors:** Hongyan Liu, Hui Gao, Jin-xia Zhao, Ut-kei Wong, Shi-bo Liu, Jian Liu, Ge Zhang, Kuan-ting Wang, Yan Wang, Lin Zhao, Xiang-bo Ma, Yue-wu Lu, Xue-wu Zhang

**Affiliations:** ^1^Department of Rheumatology and Immunology, Beijing Chao-Yang Hospital, Capital Medical University, Beijing, China; ^2^Department of Rheumatology and Immunology, Peking University International Hospital, Beijing, China; ^3^Department of Rheumatology and Immunology, Peking University Third Hospital, Beijing, China; ^4^Department of Retroperitoneal Tumor Surgery, Peking University International Hospital, Beijing, China; ^5^Department of Rheumatology and Immunology, Aerospace Center Hospital, Peking University Aerospace School of Clinical Medicine, Beijing, China; ^6^Department of Rheumatology and Immunology, Beijing Haidian Hospital, Beijing Haidian Section of Peking University Third Hospital, Beijing, China; ^7^Department of Rheumatology and Immunology, Peking University Shougang Hospital, Beijing, China; ^8^Department of Rheumatology and Immunology, The Fifth Affiliated Hospital of Zhengzhou University, Zhengzhou, China; ^9^Department of Endocrinology, Rheumatology, and Immunology, The Fourth Clinical College of Xinxiang Medical University, Xinxiang, China; ^10^Department of Rheumatology and Immunology, Handan First Hospital, Handan, China; ^11^Department of Rheumatology and Immunology, Peking University People's Hospital, Beijing, China

**Keywords:** retroperitoneal fibrosis, hydronephrosis, risk factor, poor prognosis, treat to cohort study

## Abstract

**Objective:**

Retroperitoneal fibrosis (RPF) is a rare disease characterized by the presence of fibroinflammatory tissue that surrounds the abdominal aorta and the iliac arteries and often entraps the ureters. Hydronephrosis is a common complication of RPF, however, its clinical features and outcomes have not been well elucidated.

**Methods:**

A total of 115 RPF-related hydronephrosis patients have been recruited from 9 clinical centers in China since March 2010. They were followed up until death or September 2021, whichever came first.

**Results:**

The mean age at diagnosis was 58.83 ± 12.13 years, and 80 patients (69.57%) were men. The median disease duration was 3.00 (1.00, 9.00) months. Renal impairment was observed in 88.35% of the patients, and 49.57% showed bilateral ureteral involvement. Elevated ESR and CRP were presented in 80.28 and 62.02% of the patients, respectively. Overall, 28.21% (11/39) of the patients had increased IgG4 levels, and 41.38% (12/29) showed positive pathological IgG4 staining (IgG4^+^/IgG^+^ ≥ 40% or IgG4^+^ ≥ 10/HPF). Among them, three patients were diagnosed as IgG4RD. After 60.43 ± 34.53 months of follow-up, 36 patients had poor prognosis, which was associated with severe kidney impairment, bilateral hydronephrosis and inflammation status (elevated ESR and IgG) at diagnosis by case–control study. eGFR and creatinine were independent risk factors after adjusting for all other significant associations (*p* = 0.002 and *p* = 0.067, respectively). Glucocorticoid-based therapy could reduce the time of stenting, decrease the need for long-term ureteral stenting/percutaneous nephrostomy (PNS)/ureterolysis, increase the rate of mass shrinkage, and reduce the new requirement of hemodialysis compared to surgery-only strategy for RPF-related hydronephrosis patients in need of renal drainage, but did not reduce new-onset renal atrophy.

**Conclusion:**

Severity of kidney dysfunction and inflammation status were related to the poor prognosis of hydronephrosis induced by RPF. More efficient interventions and strategies are needed to further improve outcomes.

## Introduction

Retroperitoneal fibrosis (RPF) is a rare fibroinflammatory disease characterized by fibrous tissue proliferation in the retroperitoneum (1). Ureteral involvement has been recorded in as many as 70 ~ 80% of RPF patients, which can lead to acute or chronic renal failure and even end-stage renal disease (ESRD) due to obstructive uropathy (1).

The majority of RPF are idiopathic (2, 3), although they can be secondary to infections, malignancies, drugs, retroperitoneal hemorrhage, or various other disorders. Current treatment strategies for idiopathic RPF associated with hydronephrosis are to relieve obstruction (4–6) and suppress inflammation (1). However, Chronic Kidney Disease stage 3 or worse renal function has been reported in nearly half of idiopathic RPF patients (7), which is generally associated with RPF-related hydronephrosis.

To date, outcomes have been evaluated mainly on the recurrence of RPF. Relapse occurred in 17.6 ~ 72% of the RPF patients when medication attenuated or stopped, and its predictors had been elucidated in some studies (1, 7–10). However, the factors that affect outcomes of hydronephrosis caused by RPF have been rarely discussed (7). In this study, we analyzed 115 patients with retroperitoneal fibrosis-related hydronephrosis in a multi-center cohort of the Chinese population.

## Materials and methods

### Patients

A total of 115 patients diagnosed with idiopathic RPF with renal hydronephrosis were recruited from 9 clinical centers in China between March 2010 and September 2021. According to the comprehensive diagnostic criteria for immunoglobulin G4-related disease (IgG4-RD) published by Umehara et al. (11) in 2012, patients with or without IgG4-RD were included if they were not caused by secondary conditions: definite other connective tissue disease, infections, drugs, or malignancies, etc. The study was approved by the Research Ethics Committee of the Peking University International Hospital [ethics approval number: 2019–031 (BMR)].

Information such as age, gender, disease duration, clinical manifestations, treatments, and prognosis was recorded. Medications included glucocorticoids, immunosuppressants, biologics, and other anti-fibrotic drugs. Surgical interventions included placement of ureteral stents, percutaneous nephrostomy (PNS), and ureterolysis. All patients in the study were followed up until death or September 2021, whichever came first. A poor prognosis, as defined by outcomes resulting from RPF-related hydronephrosis, includes the need for long-term ureteral stenting/ PNS/ureterolysis, failure to improve hydronephrosis, new requirement for continued hemodialysis/peritoneal dialysis, no shrinkage of mass, disease recurrence, and new renal atrophy.

### Laboratory tests, imaging, and histopathological examination

Laboratory tests comprised complete blood count (CBC), erythrocyte sedimentation rate (ESR), C-reactive protein (CRP), serum immunoglobulin (Ig) level, immunoglobulin G4 (IgG4) level, serum creatine, and estimated glomerular filtration rate (eGFR).

For those who underwent image examinations, including computed tomography (CT) or magnetic resonance imaging (MRI), the height, width, and thickness of the mass were measured. Shrinkage of mass is defined as the shortening of height, width, and thickness of the mass.

Formalin-fixed and paraffin-embedded sections were prepared and used for the histopathological examinations. Plasma cells containing IgG and IgG4 were stained by immunohistochemistry. The number of IgG4^+^ and IgG^+^ plasma cells and ratios of IgG4^+^/IgG^+^ were calculated. Diagnosis of IgG4-related RPF was defined as an IgG4^+^/IgG^+^ cell ratio >40% and more than 10 IgG4^+^ cells per high-power field.

### Statistical analysis

SPSS software, version 26, was used for statistical analyses. Continuous variables are presented as the mean (SD) for a normal distribution or the median (interquartile range: 25th–75th percentile) for a non-normal distribution. Categorical variables are presented as frequencies. An independent sample *t*-test was used for pairwise comparison between groups of continuous variables with normal distribution and equal variance. The Wilcoxon rank-sum test was performed to analyze continuous data with a non-normal distribution. Categorical variables were tested using a chi-squared test. All variables with significant differences between the good and poor prognosis groups were selected and analyzed using multiple Cox regression. A two-sided *p* < 0.05 was considered significant for all statistical tests.

## Results

A total of 115 patients from nine clinical centers diagnosed with RPF with hydronephrosis were enrolled in this study (Table 1). The mean age at diagnosis was 58.83 ± 12.13 years, and 80 patients (69.57%) were men. The median disease duration was 3.00 (1.00, 9.00) months. Back pain (46.87%) and abdominal pain (30.43%) were the most common complaints. Approximately half of the patients (49.57%) with RPF-related renal hydronephrosis showed bilateral ureteral involvement. More patients presented with left-sided hydronephrosis (29.57%) than right-sided (16.52%) in the remaining cases. Renal impairment was observed in 88.35% of the patients. Surprisingly, 59.13% of the patients had kidney function at CKD stage 3 or worse (eGFR<60 mL/min/1.73m^2^). In addition, 60% of patients had anemia. Elevated ESR and CRP were observed in 80.28 and 62.02% of these patients, respectively. In addition, 28.21% (11/39) of the patients had increased IgG4 levels, and 41.38% (12/29) showed positive pathological IgG4 staining (IgG4^+^/IgG^+^ ≥ 40% or IgG4^+^ ≥ 10/HPF). Among them, three patients were diagnosed with IgG4RD.

**Table 1:** Characteristics of RPF patients with hydronephrosis.

Characteristics	Values
Men, *n* (%)	80 (69.57%)
Age at diagnosis, years old	58.83 ± 12.13
Disease duration, months	3.00 (1.00, 9.00)
Department, *n* (%)	
Internal medicine department	40 (34.78%)
Surgical department	75 (65.22%)
Clinical manifestations	
Back pain, *n* (%)	53 (46.87%)
Abdominal pain, *n* (%)	35 (30.43%)
Lower limb edema, *n* (%)	14 (12.17%)
Abdominal distension, *n* (%)	11 (9.57%)
Nausea, *n* (%)	11 (9.57%)
Urethral irritation, *n* (%)	10 (8.70%)
Dysuria, *n* (%)	9 (7.83%)
Vomiting, *n* (%)	8 (6.96%)
Fatigue, *n* (%)	5 (4.35%)
Narration, *n* (%)	5 (4.35%)
Constipation, *n* (%)	4 (3.48%)
Fever, *n* (%)	1 (0.87%)
Hydronephrosis	
Left	34 (29.57%)
Right	19 (16.52%)
Bilateral	57 (49.57%)
Unknown	5 (4.35%)
Renal atrophy	18 (15.65%)
Renal dysfunction	
Creatinine, umol/L	196.94 ± 183.40
Increased creatinine, *n* (%)	88 (76.5%)
e-GFR, ml/min/1.73m^2^	54.26 ± 31.94
Decreased eGFR, *n* (%)	97 (88.35%)
CKD stage 1, *n* (%)^a^	18 (16.65%)
CKD stage 2, *n* (%)	29 (25.22%)
CKD stage 3, *n* (%)	36 (31.30%)
CKD stage 4, *n* (%)	21 (18.26%)
CKD stage 5, *n* (%)	11 (9.57%)
ESR, mm/h	46.20 ± 30.89
Increased ESR, *n* (%)	57/71 (80.28%)
C-reactive protein, mg/dl	14.92 ± 32.75
Increased CRP, *n* (%)	49/79 (62.02%)
Hemoglobin, g/L	110.72 ± 24.14
Decreased HGB, *n* (%)	69/115 (60.00%)
IgG, g/L	14.76 ± 4.51
Increased IgG, *n* (%)	25/72 (34.72%)
IgG4, g/L	2.14 ± 3.03
Increased IgG4, *n* (%)	11/39 (28.21%)
Positive pathological IgG4 staining, *n* (%)^b^	12/29 (41.38%)

^a^CKD stage 1: eGFR ≥ 90 mL/min/1.73m^2^; CKD stage 2: eGFR60-89 mL/min/1.73m^2^; CKD stage 3: eGFR30-59 mL/min/1.73m^2^; CKD stage 4: eGFR15-29 mL/min/1.73m^2^; CKD stage 2: eGFR<15 mL/min/1.73m^2^; ^b^IgG4+/IgG + ≥40% or IgG4 + ≥10/HPF.

**Figure 1:**
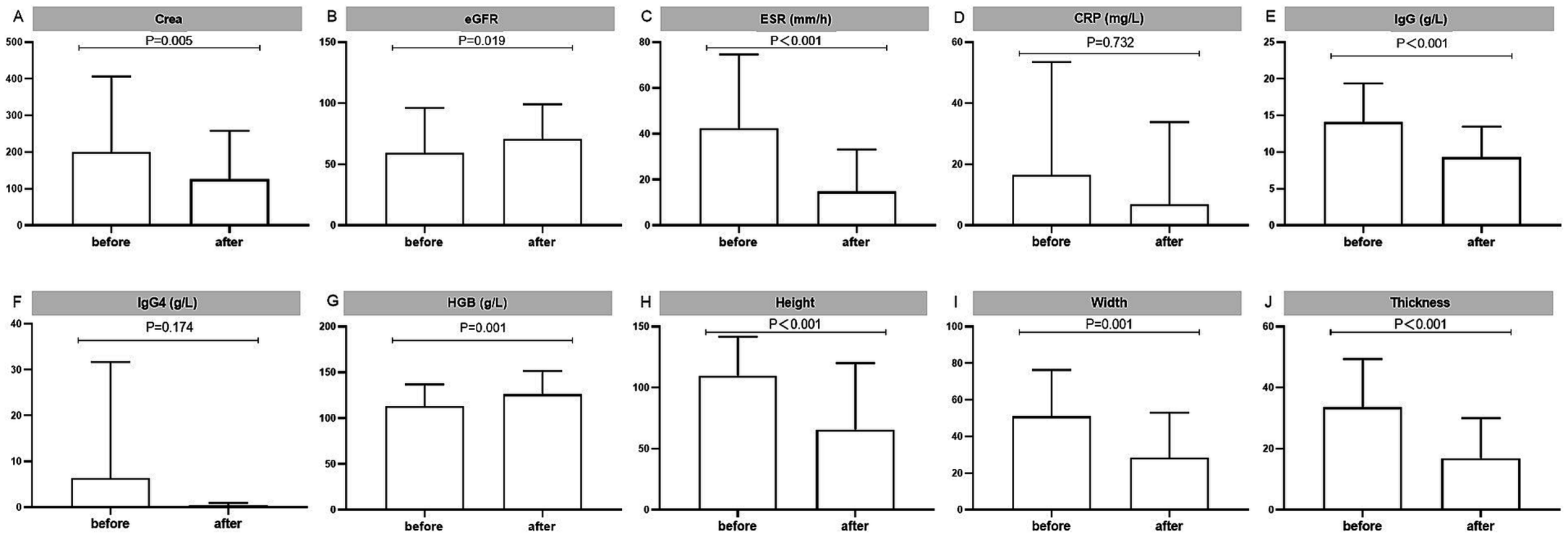
Laboratory tests to assess the treatment outcomes of the RPF patients. Displayed are **(A,B)** kidney functions, **(C,D)** inflammatory markers, **(E)** IgG and **(F)** IgG4, **(G)** hemoglobin, and **(H–J)** changes of fibrous tissue mass, as measured in 63 patients with data before and after treatment. Ig, immunoglobulin.

A total of 94 (81.74%) RPF patients with hydronephrosis received renal drainage, which was not required for the rest because of mild disease severity (Table 2). Stent implantation (67.83%) was the most common intervention. However, 13.43% of the patients needed 2 or more kinds of surgical treatments. Ureterolysis and percutaneous nephrostomy were performed in 11.30 and 7.83% of the patients, respectively. Glucocorticoid-based therapy was prescribed for 71.30% of the patients, and 33.40% of the patients were given corticosteroid-sparing agents (Table 2). Interestingly, the chosen medicine varied widely, including tamoxifen (13.04%), cyclophosphamide (11.30%), sirolimus (6.96%), mycophenolate mofetil (4.35%), azathioprine (3.48%), methotrexate (3.48%), and hydroxychloroquine (3.48%). A total of 12.17% of the patients needed 2 or more kinds of corticosteroid-sparing agents. Kidney function (serum creatine and eGFR), anemia, hyperglobulinemia (IgG), and inflammatory status (ESR) improved significantly after treatment (Figure 1).

**Table 2:** Interventions for RPF patients with hydronephrosis.

Treatment	Values
Glucocorticoids	82 (71.30%)
Corticosteroid-sparing agents	38 (33.40%)
Tamoxifen	15 (13.04%)
Cyclophosphamide	13 (11.30%)
Sirolimus	8 (6.96%)
Mycophenolate mofetil	5 (4.35%)
Hydroxychloroquine	4 (3.48%)
Azathioprine	4 (3.48%)
Methotrexate	4 (3.48%)
Rituximab	3 (2.61%)
Leflunomide	2 (1.74%)
Tripterygium glycosides	2 (1.74%)
Tacrolimus	1 (0.87%)
≥2 kinds of drugs (Glucocorticoid not included)	14 (12.17%)
Surgical treatment, *n* (%)	94 (81.74%)
Stent implantation	78 (67.83%)
Ureterolysis	13 (11.30%)
Percutaneous nephrostomy	9 (7.83%)
Unknown surgery	13 (11.30%)
≥2 kinds of surgical treatment	15 (13.43%)

According to the criteria described in the “Methods” section, 36 patients had a poor prognosis after 60.43 ± 34.53 months of follow-up. Sex, age, and disease duration were comparable between the two groups (Table 3). Severe kidney impairment and bilateral hydronephrosis at diagnosis were risk factors for poor prognosis, especially those with worse renal disease (eGRF<60 mL/min/1.73m^2^, 51.90 vs. 75%, *p* = 0.019). In addition, inflammation status of elevated ESR, hyperglobulinemia (IgG), and anemia were also associated with poor prognosis (Table 3). After adjustment in a multivariable Cox regression model, the eGFR and level of creatinine at diagnosis were regarded as prognostic factors (*p* = 0.002 and *p* = 0.067 respectively), rather than the level of ESR, CRP, IgG, HGB, or hydronephrosis. Although medical and surgical interventions were not related to prognosis in RPF patients with renal hydronephrosis, glucocorticoid-based therapy could reduce the need for long-term ureteral stenting/ PNS/ureterolysis (29.51 vs. 60.87%, *p* = 0.001, Table 4) and decrease the time of stenting [10.00 (6.00, 8.00) months vs. 36.00 (12.00, 97.00) months, *p* = 0.014] (Table 4) for hydronephrosis patients in need of renal drainage. In addition, hydronephrosis patients treated with glucocorticoid also benefited from medical treatment in terms of significant mass shrinkage (70.5 vs. 15.2%, *p* < 0.001) and low rate of hemodialysis (4.9 vs. 12.1%, *p* = 0.391). However, more patients in this subgroup developed new renal atrophy during the follow-up (11.5 vs. 3.0%, *p* = 0.038). More efficient interventions and strategies are needed to further improve outcomes.

**Table 3:** Risk factors of poor prognosis in RPF patients with hydronephrosis.

Characteristics	Good prognosis (*N* = 79)	Poor prognosis (*N* = 36)	*P*
Male sex, *n* (%)	56 (70.89%)	24 (66.67%)	0.648
Age at diagnosis, years	59.09 ± 11.93	58.27 ± 12.71	0.747
Disease duration, months	10.25 ± 19.66	5.60 ± 7.79	0.175
Hydronephrosis			
Unilateral	43 (54.43%)	10 (27.78%)	0.002
Bilateral	31 (39.24%)	26 (72.22%)	0.002
Unknown	5 (6.33%)	0	–
Renal atrophy	11 (13.92%)	7 (19.44%)	0.281
Renal dysfunction			
e-GFR, ml/min/1.73 m2	59.46 ± 32.03	42.85 ± 29.00	0.007
CKD ≥ stage 3, *n* (%)^a^	41/79 (51.90%)	27/36 (75.00%)	0.019
Creatinine, umol/L	170.21 ± 158.08	255.61 ± 220.72	0.042
ESR, mm/h	39.43 ± 29.40	61.27 ± 29.31	0.005
Increased ESR, *n* (%)	36/49 (73.47%)	21/22 (95.45%)	0.031
C-reactive protein, mg/dl	2.45 ± 3.69	3.14 ± 3.02	0.414
Increased CRP, *n* (%)	31/53 (58.49%)	18/26 (69.23%)	0.355
Hemoglobin, g/L	113.13 ± 25.48	105.43 ± 20.24	0.113
Decreased HGB, *n* (%)	42/79 (53.16%)	27/36 (75.00%)	0.027
IgG, g/L	14.45 ± 4.62	15.41 ± 4.30	0.396
Elevated IgG, *n* (%)	13/49 (26.53%)	12/23 (52.17%)	0.033
IgG4, g/L	2.43 ± 3.60	1.58 ± 1.34	0.402
Elevated IgG4, *n* (%)	6/25 (24.00%)	5/14 (35.71%)	0.435
Positive pathological IgG4, *n* (%)^b^	7/15 (46.67%)	5/14 (35.71%)	0.924
Treatment			
Glucocorticoids (with or without surgery)	59 (74.68%)	23 (63.89%)	0.235
Surgery (without glucocorticoids)	20 (25.32%)	13 (36.11%)	0.235

^a^CKD stage ≥ 3: CKD 3, CKD 4, and CKD 5, eGFR<60 mL/min/1.73m^2^. ^b^IgG4+/IgG + ≥40% or IgG4 + ≥10/HPF.

**Table 4:** Outcomes of RPF-related hydronephrosis patients in need of renal drainage.^#^

Characteristics	Surgical treatment (*N* = 33)	Combined treatment (*N* = 61)	*P*-value
New-onset renal atrophy, *n* (%)	1 (3.0%)	7 (11.5%)	0.038
Shrinkage of mass, *n* (%)	5 (15.2%)	43 (70.5%)	<0.001
Long-term ureteral stenting/PNS/ureterolysis, *n* (%)	14 (60.87%)	18 (29.51%)	0.001
Time of ureteral stenting, months	36.00 (12.00, 97.00)	10.00 (6.00, 8.00)	0.014
Hemodialysis, *n* (%)	4 (12.1%)	3 (4.9%)	0.391

^#^A total of 94 (81.74%) RPF patients with hydronephrosis were given renal drainage and analyzed in this table.

## Discussion

In this study, we described the demographic, clinical, laboratory, imaging, and therapeutic features of 115 RPF patients with hydronephrosis in a multi-center cohort study of Chinese patients, as well as the prognosis after the intervention. Then, we explored risk factors for poor prognosis of hydronephrosis, which was found to be related to severe kidney impairment, bilateral hydronephrosis, and inflammation status of elevated ESR and IgG at diagnosis by a case–control study. eGFR and creatinine were identified as independent correlates after adjusting for all other significant associations. Finally, we found that glucocorticoid-based therapy could reduce the time of stenting, decrease the need for long-term ureteral stenting/ PNS/ ureterolysis, increase the rate of mass shrinkage, and reduce the rate of hemodialysis, but did not reduce new-onset renal atrophy. More efficient interventions and strategies are needed to further improve the outcomes of RPF patients with hydronephrosis.

RPF is a rare disease, and the idiopathic form is found in more than two-thirds of all cases (1). The remaining cases are secondary to different causes, such as drugs, infections, tumors, trauma, and radiotherapy (1, 4). Studies have demonstrated that ≤50% of idiopathic RPF cases are associated with IgG4-related diseases (2, 12). IgG4-related and -unrelated RPFs have similar demographic and laboratory characteristics, comparable mass location and thickness, and almost identical rates of ureteral involvement (12). Therefore, we recruited both IgG4-related and -unrelated RPFs.

Idiopathic retroperitoneal fibrosis is reported with an estimated prevalence of 1.4 cases/100,000 inhabitants (3). The male-to-female ratio is 2:1–3:1, and the mean age at onset ranges between 55 and 60 years (13). Ureteral obstruction occurs in approximately 60 ~ 90% of cases and often causes acute renal failure (14, 15). RPF patients with ureteral obstruction and hydronephrosis are prone to develop CKD, ESRD, and kidney atrophy (16). In this study, the prevalence of hydronephrosis, as well as the age and sex proportion, was comparable to those reported in previous studies. CKD stage 3 or worse renal function occurred in more than half of our patients (59.13%) at diagnosis, which is more severe than that reported in previous studies (7). In addition, 20% of the RPF-related hydronephrosis patients had a poor prognosis during follow-up. However, the exact risk factors have not been well-elucidated before. Our study showed that severe kidney impairment, bilateral hydronephrosis, and inflammation status (elevated ESR and IgG) at diagnosis were associated with poor prognosis. However, only severe kidney dysfunction (eGFR and creatinine) was regarded as an independent risk factor. Thus, prompt diagnosis at an early stage and efficient intervention to improve hydronephrosis and renal dysfunction are crucial for improving outcomes of hydronephrosis in RPF.

Treatment strategies for idiopathic RPF have been rather empirical and varied widely in clinical practice (16–26). Glucocorticoid-based medical treatment has been introduced to avert the progression and recurrence of fibrosis caused by inflammation (1). Stent implantation, PNS, and ureterolysis are common strategies to relieve ureteral obstruction (4–6). The success rate of ureteral stenting at the first step was reported to be successful only in 69–79% of the patients (5, 27). The need for using two or three surgical options in the same patient might be required over time (28). In this study, 14.3% of the patients needed more than 2 kinds of renal drainage. More than half (60.87%) of the patients treated with renal drainage alone needed long-term ureteral stenting/ PNS/ ureterolysis.

Medical treatment has been used to avert progression of the fibrosis caused by inflammation (1). Tamoxifen was a first-generation drug of RPF for its anti-fibroblastic effects (21, 28). Later in 2011, Vaglio et al. (1) reported in a randomized clinical trial that glucocorticoid monotherapy achieved better treatment outcomes than tamoxifen (25) and has since become the mainstay therapy for RPF. However, glucocorticoid monotherapy had limited effect as an initial treatment and a high relapse rate after its attenuation or discontinuation (1, 7, 27–29). Many immunosuppressants have been introduced, including methotrexate (26), mycophenolate mofetil (22, 23), and cyclophosphamide (18). However, no immunosuppressant has been shown to be superior to other options (27). We leveraged bioinformatic analysis to systematically interrogate the biological mechanisms of RPF and inferred that sirolimus was a potential drug for treating it (29). We then designed a therapy combining a gradual reduction of prednisone with a long-term stable dosage of sirolimus, which resulted in good efficacy and tolerance. Future controlled studies are needed to determine its superiority to traditional glucocorticoid monotherapy.

## Conclusion

In this study, glucocorticoid-based medical treatment reduced the time of stenting, decreased the need for long-term ureteral stenting/ PNS/ureterolysis, increased the rate of mass shrinkage, and reduced the rate of hemodialysis compared to the surgery-only group. However, a greater proportion of patients treated with medication combined with renal drainage developed new renal atrophy, although the majority of the patients presented improvement in hydronephrosis after treatment. Our previous study showed that the onset of RPF is insidious and lacks specific initial symptoms (30). More importantly, hydronephrosis can even develop before the onset or recurrence of symptoms. Abdominal ultrasound at a regular checkup revealed hydronephrosis without any other symptoms in approximately 10% of the patients (31). The persistent presence of hydronephrosis may be associated with kidney atrophy (7). duration of ureteral stent placement in medication combined renal drainage group of this study may increase the insidious recurrence of hydronephrosis, which should be explored in future prospective studies. More efficient interventions and strategies, such as earlier ureterolysis and closer monitoring of the urinary tract through ultrasound, are needed to further improve renal outcomes.

There are several limitations in the current study. First, as a retrospective study, laboratory tests and imaging were not thoroughly assessed, especially those from the surgery department. Second, IgG4 staining and serum IgG4 tests were not routine pathological tests a decade ago, so it is not accurate to evaluate IgG4-related RPF in this study. Third, as a retrospective cohort study, the causal relationships observed in this study should be further evaluated in a large prospective study.

## Data Availability

The original contributions presented in the study are included in the article/Supplementary material, further inquiries can be directed to the corresponding authors.
