# Supplementary material for: Outcomes of retroperitoneal fibrosis-related hydronephrosis and its risk factors for poor prognosis: a multi-center retrospective cohort study in Chinese patients
Source: Front Med (Lausanne). 2024 Dec 9;11:1435870. doi: 10.3389/fmed.2024.1435870 (PMC11663637; doi:10.3389/fmed.2024.1435870)
Supplement: Supplementary file 1 [file Table_1.DOCX]

Supplementary Table 1. Comparation of laboratory tests before and after treatment in RPF patients with hydronephrosis ^#^

|  | before treatment | after treatment | *P* |
| --- | --- | --- | --- |
| Creatine, umol/L | 221.52±214.63 | 128.49±141.46** | 0.005 |
| e-GFR, ml/min/1.73m^2^ | 52.43±34.60 | 65.70±27.37* | 0.019 |
| ESR, mm/h | 48.2±32.39 | 16.45±19.99** | ＜0.001 |
| C-reactive protein, mg/dl | 1.48(0.50,4.92) | 0.22(0.08,0.65)** | ＜0.001 |
| Hemoglobin, g/L | 109.48±21.71 | 123.92±26.40** | 0.001 |
| IgG, g/L | 14.59±5.52 | 9.95±4.09** | ＜0.001 |
| IgG4, g/L | 0.89(0.50,1.84) | 0.45(0.25,0.84) ** | ＜0.001 |
| Height, mm | 111.45±31.98 | 63.52±55.59** | ＜0.001 |
| Width, mm | 51.68±26.51 | 27.74±25.48** | 0.001 |
| Thickness, mm | 33.61±16.63 | 16.39±13.39** | ＜0.001 |

# A total of 63 patients with data before and after treatment were collected and compared in this table. The levels of CRP and IgG4 were presented median (quartile 1, quartile 3), others presented mean ±s.

* compared with before treatment, *p*＜0.05; ** compared with before treatment, *p*＜0.01.
